# Sutureless technique versus conventional surgery in the primary treatment of total anomalous pulmonary venous connection: a systematic review and meta-analysis

**DOI:** 10.1186/s13019-018-0756-z

**Published:** 2018-06-15

**Authors:** Yuhao Wu, Zhichao Wu, Junmeng Zheng, Yonggang Li, Yuehang Zhou, Hongyu Kuang, Xin Jin, Chun Wu

**Affiliations:** 1 Department of Cardiothoracic Surgery, Children’s Hospital of Chongqing Medical University, No.136 Zhongshan Second Road, Yuzhong District, Chongqing, 400014 China; 2 Department of Cardiovascular Surgery, Sun Yat-Sen Memorial Hospital, Sun Yat-Sen University, No.107 Yanjiang West Road, Yuexiu District, Guangzhou, 510120 China; 3 Department of Cardiology, Children’s Hospital of Chongqing Medical University, Chongqing, 400014 China; 4 Ministry of Education Key Laboratory of Child Development and Disorders, Chongqing, 400014 China; 5 China International Science and Technology Cooperation Base of Child Development and Critical Disorders, Chongqing, 400014 China; 6 Chongqing Key Laboratory of Pediatrics, Chongqing, 400014 China

**Keywords:** Congenital heart disease, Total anomalous pulmonary venous connection, Sutureless technique, Conventional surgery, Meta-analysis

## Abstract

**Backgroud:**

A meta-analysis was performed to compare the differences in outcomes between sutureless technique and conventional surgery for primary repair of Total Anomalous Pulmonary Venous Connection(TAPVC).

**Methods:**

Electronic databases, including PubMed, EMbase, Medline, CNKI, Wanfang Data and Weipu Data were searched systematically for the literature aimed mainly at comparing the therapeutic effects for primary repair of TAPVC administered by sutureless technique and conventional surgery. Corresponding data sets were extracted and two reviewers independently assessed the methodological quality.

**Results:**

Seven studies meeting the inclusion criteria were included, involving a total of 1293 subjects. It was observed that sutureless technique entailed a lower occurrence rate of post-operative Pulmonary Veins Obstruction (PVO) (OR, 0.52 95%CI, 0.32–0.86; *P* = 0.01) and re-operation due to PVO (OR, 0.28;95%CI, 0.09–0.87; *P* = 0.03). However, meta-analyses of hospitalization time (WMD, 5.92; 95%CI, − 7.97-19.80; *P* = 0.40) and post-operative mortality (OR, 0.65; 95%CI, 0.41–1.04; *P* = 0.07) showed no significant differences between sutureless technique and conventional surgery. Meta-analysis of Cardiopulmonary Bypass (CPB) time and aortic cross-clamp time also showed no significant differences between the two surgical approaches (WMD, 5.07; 95%CI, − 9.29-19.42; *P* = 0.49); (WMD, 5.73; 95%CI, − 7.76-19.23; *P* = 0.40), but the result remained inconclusive due to pooling result changes after sensitivity analysis.

**Conclusions:**

Compared with conventional surgery, a lower occurrence rate of post-operative PVO and re-operation due to PVO were associated with sutureless technique. Meanwhile, hospitalization time and post-operative mortality were not statistically different between the two surgical approaches. Pooling result of CPB and aortic cross-clamp time between the two groups remained inconclusive.

## Background

Total Anomalous Pulmonary Venous Connection (TAPVC), a common congenital heart defect in which four pulmonary veins do not connect to the left atrium directly, occurres to 5.9–7.1 newborns per 100,000 neonates [1]. Traditionally, the main causes of death after conventional surgery for TAPVC are pulmonary hypertension and post-operative Pulmonary Venous Obstruction (PVO) [2]. In the 1990s, sutureless technique was employed to relieve the post-operative PVO [3, 4], in which the direct anastomosis between the left atrium and pulmonary veins was absent by creating a new left atrium by means of anastomosing the left atrium to the posterior pericardium. In recent years, this sutureless technique, utilized also for the primary repair of TAPVC, has been expanded for use in patients who had preoperative PVO or were at risk of developing PVO [5].

Although sutureless technique for primary repair of TAPVC was conducted by many advanced children’s medical center, the safety and efficacy of sutureless technique remained controversial. This meta-analysis aimed to evaluate the outcomes of sutureless technique and conventional surgery for primary repair of TAPVC, as well as to provide explicit evidence to determine if sutureless technique in the treatment of TAPVC was superior to conventional surgery.

## Methods

### Literature search

We conducted the systematic search on the PubMed, EMbase, Medline, CNKI, Wanfang Data and Weipu Data for the relevant studies comparing the outcomes of applying sutureless technique and conventional surgery for priamry repair of TAPVC. The key words used for the search were *sutureless technique, sutureless surgery, conventional surgery, surgical repair, total anomalous pulmonary venous connection* and *total anomalous pulmonary venous drainage*. References from all the included studies and other relevant literature were also manually reviewed to identify additional eligible studies. This search was restricted to articles published in English and Chinese. The time period for the search was from January 1998 to March 2018. We contacted the authors through e-mail to acquire additional information as necessary.

### Study selection

A study was included in this systematic review if it met both criteria as follow: 1) Cohort or case-controlled studies or Randomized Controlled Trials (RCTs); 2) The comparison of sutureless technique and conventional surgery for primary repair of TAPVC should be described in details. Specifically for sutureless technique, an incision was made on the posterior wall of the left atrium and the left atrial was anastomosed with the pericardium adjacent to the pulmonary vein, avoiding direct anastomosis with the pulmonary venous wall. For conventional surgery, the common pulmonary vein confluence was anastomosed to the left atrium directly for the supra-, infra- cardiac and mixed TAPVC. In the case of intracardiac TAPVC, an intra-atrial baffle technique between the atrial septal defect and the coronary sinus was created.

A study was excluded fromthis systematic review if any of the following was true: 1) Multiple studies were based on the same data; 2) Studies included sutureless technique for recurrent or post-operative PVO; 3) Studies only included patients with congenital or secondary pulmonary vein stenosis. 4) The clinical outcomes were not to our interest. Reviews, conference records, case reports and animal experiments were also excluded.

Two reviewers (W.Y.H and W.Z.C) screened all the studies independently and blindly. Any disagreements on the eligibility of studies were resolved by discussion with the third reviewer(K.H.Y).

### Data extraction

Data extracted by two reviewers (W.Y.H and W.Z.C) independently and blindly were checked by the third reviewer(K.H.Y) for accuracy. A standardized extraction form in an Excel spreadsheet was used. The following information was extracted: 1) baseline characteristics of included studies: first author, publication year, study design, surgical approach, sample size, type of TAPVC, weight, age, occurrence of preoperative PVO, associated cardiac anomalies and follow-up time; 2) outcomes of both surgical approaches: cardiopulmonary bypass time, aortic cross-clamp time, post-operative mortality, total number of operation, occurrence rate of post-operative PVO and re-operation. Post-operative death was defined as death related to the surgery directly. Post-operative PVO was defined as obstruction in the anastomosis that occurred after initial surgery and diagnosed with cardiac echo. Re-operation was defined as surgery performed only for post-operative PVO that occurred after discharge. Total number of operation was defined as the sum of initial surgery plus re-operation.

### Quality assessment

Quality assessment was performed by two reviewers (W.Y.H and W.Z.C) independently and bindly. Any disagreements on the assessed quality were resolved via consultation with the third reviewer(K.H.Y). The quality of the included studies was evaluated by using Newcastle-Ottawa Scale (NOS) scores. A study was judged on three dimensions with the Newcastle-Ottawa Scale (NOS) scale: the selection of the study groups; the comparability of the groups; and the ascertainment of the exposure [6].The maximum score of NOS was 9 and the minimum score was zero. Each study was graded as either low (scores 0–5) or high quality (scores 6–9).

### Statistical analysis

All statistical analyses were conducted by using RevMan software (version 5.3; The Nordic Cochrane Centre, Copenhagen, Denmark), and *P* < 0.05 was considered statistically significant. Odds Ratio (OR) and Weighted Mean Difference (WMD) were adopted for dichotomous and continuous data. Heterogeneity was evaluated with the Cochrane Q test and the I^2^ statistics, with I^2^ > 50% indicating heterogeneity. If the I^2^ statistic > 50%, a random-effects model was adopted, and sensitivity analysis was used to detect the sources of heterogeneity; otherwise, a fixed-effects model of analysis was adopted. Sensitivity analyses were performed using the leave-one-out method in which the meta-analysis of outcomes was conducted with each study removed in turn until significant reduction in heterogeneity was observed. If the direction and magnitude of pooling estimates with respect to outcomes did not vary significantly with the removal of any given study, it indicated that the meta-analysis was confirmed and the data were not overly influenced by any study. Formulas provided by Hozo et al. [7] would be used to estimate the mean values and standard differences if only the median value and range were available. The publication bias was evaluated with Begg’s test and Egger’s test using Stata 12.0 (Stata Corp,Texas).

## Results

**Fig. 1:**
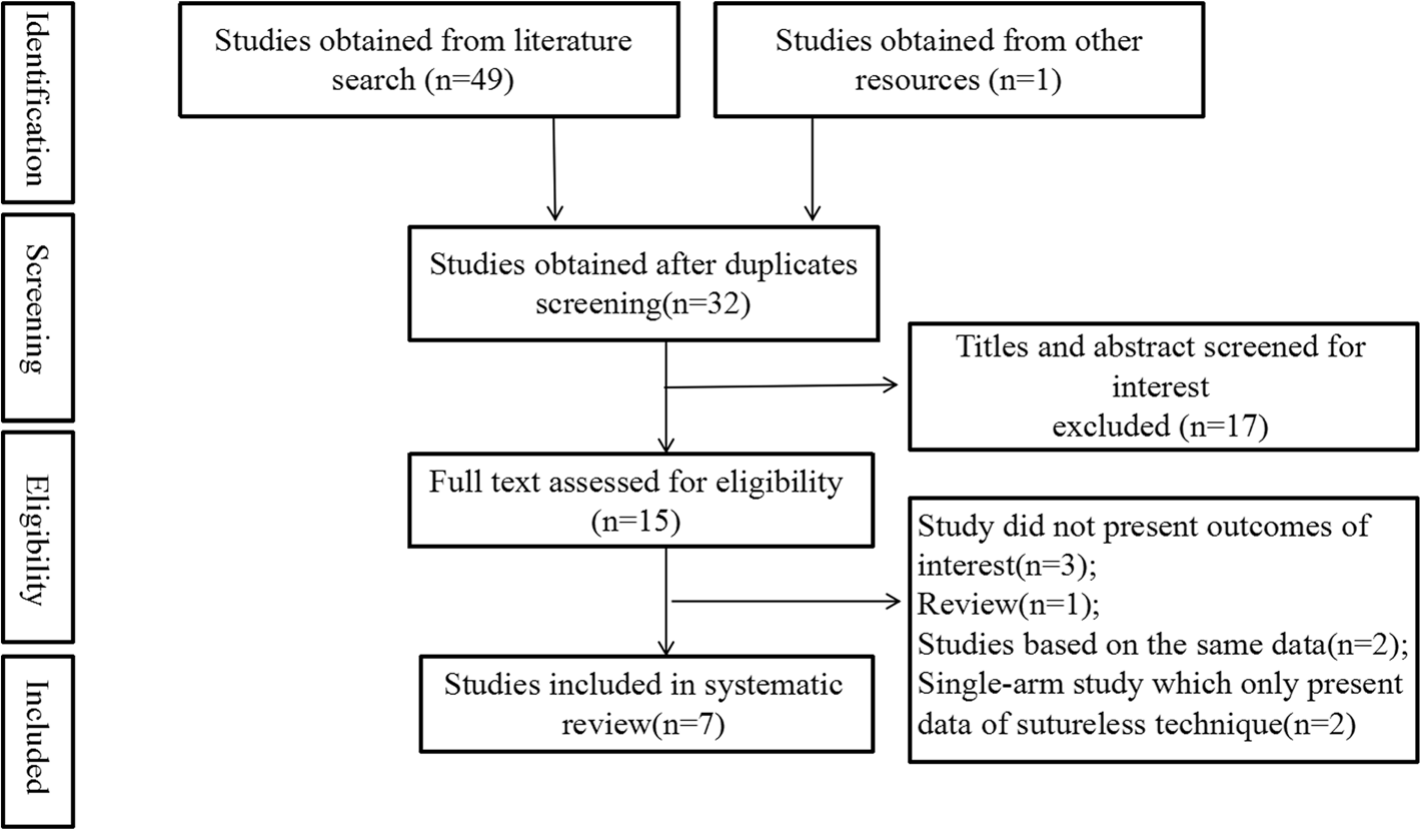
Flowchart of process for literature screening for this meta-analysis

A total of 50 studies were identified initially. After screening for duplicates and relevance in titles and abstracts, only 15 studies were eligible for the full-text evaluation. Eventually, this meta-analysis was based on 7 retrospective None-Randomized Controlled Trials(NRCTs). The literature search and study selection have been double checked by both reviewers (W.Y.H and W.Z.C), and the flowchart depicting the search strategy is shown in the Fig. 1.

### Characteristics of included studies and quality assessments

All 7 studies [8–14] were retrospective NRCTs. A Total of 1293 patients were involved in this study, of whom 278 were in the sutureless technique group and 1015 in the conventional surgery group. Quality assessments of the included studies were carried out in accordance with the NOS. The NOS score of each of the 7 included studies [8–14] was higher than 6 (high quality). The characteristics of the included studies and quality assessments were described in detail in Table 1. All of the TAPVC patients were diagnosed with cardiac echo and combined with enhanced CT scan. The associated anomalies were shown in the Table 2.

**Table 1:** Characteristics and quality assessment of included studies

Author	Publication year	Study design	Surgical approach	Patients M/F (sex)	Type of TAPVC (supracardiac/car-diac/infracar-diac/mixed)	Weight (Kg)	Age	Preoperative PVO (n)	Follow-up time	NOS
Gao [8]	2017	Retrospective	ST CS	56/25 74/24	63/0/14/4 69/0/11/18	4.3(3.3–5.3)^b^ 4.6(3.4–6)	60(25.5–150)d 60(29.3–180)d	24 28	12 m^a^ 12 m	7 (High)
Cui [9]	2016	Retrospective	ST CS	14 46	NA	3.5 ± 0.8 4.4 ± 1.2	1.8 ± 0.4 m 2.4 ± 2.2 m	NA	46 m^a^ 46 m	6 (High)
Shi [10]	2017	Retrospective	ST CS	78 690	59/2/13/4 289/278/73/50	NA	323.7 ± 23.8d 202.6 ± 41d	35 157	23.2(1–112)m 23.2(1–112)m	7 (High)
Yamashita [11]	2013	Retrospective	ST CS	5/2 4/1	4/0/2/1 3/1/1/0	3.4(3–8) 3.6(2.6–4.3)	119(55–425)d 93(42–147)d	6 2	3.9 ± 2.8y 7.2 ± 5.2y	6 (High)
Yanagawa [12]	2011	Retrospective	ST CS	12/9 20/16	12/4/5/0 19/11/6/0	3.3(1.9–5) 3.5(1.7–11.7)	11(1–128)d 19(1–1157)d	17 18	21.1(0.2–104.8)m 45.8(0–143.1)m	7 (High)
Honjo [13]	2010	Retrospective	ST CS	2/6 2/12	0/0/0/8 0/0/0/14	3.8 ± 0.6 4.7 ± 3.2	33 ± 26 d 70 ± 81 d	7 8	50 ± 56 m 121 ± 110 m	6 (High)
Lo Rito [14]	2015	Retrospective	ST CS	37/32 81/45	32/6/16/15 59/33/20/14	3.6(3.1–4) 3.6(3.1–4.6)	18(2–52)d 36(4–85)d	33 44	6.4y^c^ 6.4y	7 (High)

*a* median value *b* median value and range *c* mean value

*d* days, *m* months, *y* years

*ST* Sutureles technique group, *CS* Conventional surgery group, *NOS* Newcastle-Ottawa scale, *NA* Not available

**Table 2:** The associated anomalies of included TAPVC patients

Study	Associated anomalies(n)
Gao [8]	NA
Cui [9]	2 SV anatomy/PA stenosis; 2 unroofed coronary sinus syndrome; 1 DORV; 1 CoA; 1 VSD
Shi [10]	568 PDA; 19 VSD; 2 CoA; 1 TOF; 52 PA stenosis;
Yamashita [11]	8 PA stenosis;1 PV atresia/VSD; 1 Cor triatriatum; 1 Coronary artery fistula
Yanagawa [12]	NA^a^
Honjo [13]	2 DORV; 2 Cor triatriatum; 1 PV atresia/VSD; 1 RVOTO
Lo Rito [14]	5 VSD; 3 CoA; 3 DORV; 2 TA; 2 AVSD

^a^ This study was based on isolated TAPVC and patients associated with cardiac lesions were excluded

*SV* Single ventricle anatomy, *PA* Pulmonary artery, *DORV* Double-outlet right ventricle, *CoA* Coarctation of the aorta, *TOF* Trilogy of fallot, *PV* Pulmonary vein, *RVOTO* Right ventricular outflow tract obstruction, *VSD* ventricular septal defect, *TA* Truncus arteriosus, *AVSD* Atrioventricular septal defect, *NA* Not available

**Fig. 2:**
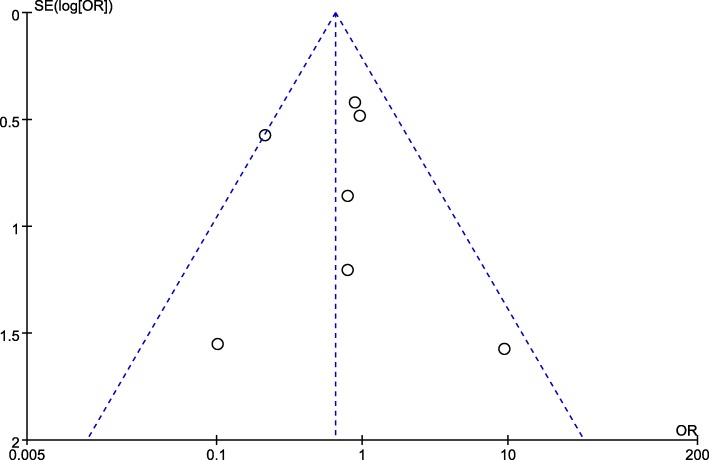
Publication bias analysis by funnel plot

The results of funnel plot could be considered accurate only when a minimum of 7 studies was included. Post-operative mortality were provided by all 7 NRCTs, therefore, we only conducted the funnel plot of post-operative mortality and no significant publication bias was found (Begg’s test *P* = 0.88, Egger’s test *P* = 0.248) (Fig. 2).

### Cardiopulmonary bypass time (CPB)

**Fig. 3:**
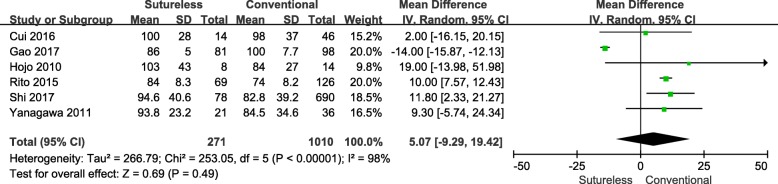
Forest plot of meta-analysis of CPB time

Six of the 7 studies [8–10, 12–14] that analyzed the CPB time of both surgical approaches were included in this meta-analysis. Since a heterogeneity test revealed Chi^2^ = 253.05, *P* < 0.001, I^2^ = 98%, therefore a randomized effects model was employed (Fig. 3). The result indicated that no statistically significant difference was observed between the two surgical approaches with respect to CPB time(WMD, 5.07; 95%CI, − 9.29-19.42; *P* = 0.49).

### Aortic cross-clamp time

**Fig. 4:**
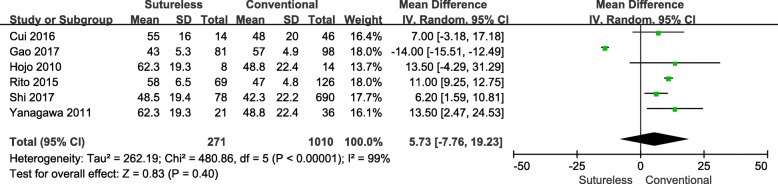
Forest plot of meta-analysis of aortic cross-clamp time

A total of 6 studies [8–10, 12–14] that analyzed the aortic cross-clamp time of both surgical approaches were included in this meta-analysis. A heterogeneity test revealed Chi^2^ = 480.06, *P* < 0.001, I^2^ = 99%, therefore a randomized effects model was employed (Fig. 4). The result indicated that no statistically significant difference was observed with regard to aortic cross-clamp time (WMD, 5.73; 95%CI, − 7.76-19.23; *P* = 0.40).

### Post-operative mortality

**Fig. 5:**
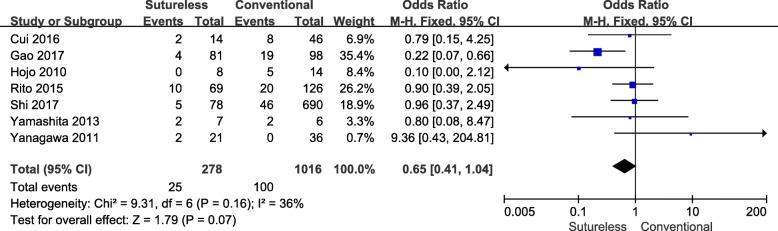
Forest plot of meta-analysis of post-operative mortality

All 7 studies [8–14] that analyzed the post-operative mortality of both surgical approaches (Table 3) were included in this meta-analysis. A heterogeneity test revealed Chi^2^ = 9.31, *P* = 0.16, I^2^ = 36%, so a fixed effects model was employed (Fig. 5). The test result indicated that no statistically significant difference existed between the two surgical approaches (OR, 0.65; 95%CI, 0.41–1.04; *P* = 0.07).

**Table 3:** Post-operative death of included studies

Study	Post-operative death(n)
Gao [8]	NA^a^
Cui [9]	1 SVC thrombosis; 1 LCOS; 8 PVO
Shi [10]	10 LCOS; 10 PAH; 5 respiratory failure; 4 MODS; 1 intracranial hemorrhage; 1 sepsis; 10 PVO; 4 death with unknown causes; 2 none-cardiac death; 4 failed to recover from bypass
Yamashita [11]	3 PVO; 1 bleeding
Yanagawa [12]	1 none-cardiac death; 1 PVO
Honjo [13]	2 sudden cardiac arrest; 2 PVO; 1 LCOS
Lo Rito [14]	4 LCOS; 3 refractory arrhythmia; 3 PAH; 1 PE; 1 sepsis; 4 PVO; 5 comorbidities without evidence of PVO

^a^ Only the numbers of death was available and the details of post-operative death were absent

*SVC* superior vena cava, *LCOS* Low cardiac output syndrome, *PVO* Pulmonary vein obstruction, *PAH* Pulmonary artery hypertension, *MODS* Multiple organs dysfunction syndrome, *PE* Pulmonary artery embolism, *NA* Not available

### Hospitalization time

**Fig. 6:**
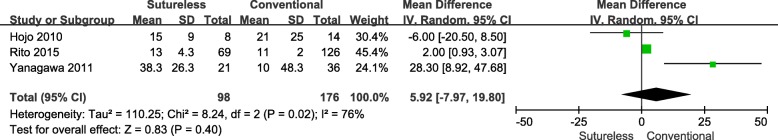
Forest plot of meta-analysis of hospitalization time

Three out of 7 studies [12–14] that analyzed the hospitalization time of both surgical approaches were included in this meta-analysis. A heterogeneity test revealed Chi^2^ = 110.25, *P* = 0.02, I^2^ = 76%, so a randomized effects model was employed (Fig. 6). This result indicated that no statistically significant difference was observed between the two surgical approaches regarding to the hospitalization time(WMD, 5.92; 95%CI, − 7.97-19.80; *P* = 0.40).

### Post-operative obstruction (PVO), re-operation and total number of operation

**Fig. 7:**
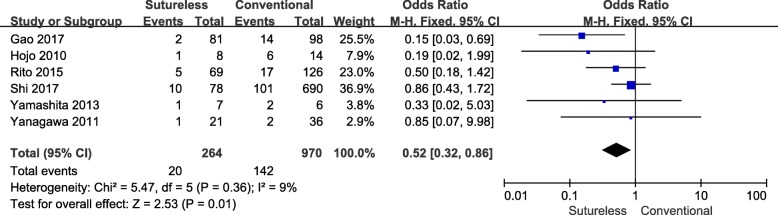
Forest plot of meta-analysis of occurrence rate of post-operative PVO

A total of 6 studies [8, 10–14] analyzed the post-operative obstruction of both surgeries (Table 4). A heterogeneity test revealed Chi^2^ = 5.47, *P* = 0.36, I^2^ = 9%, and a fixed effects model was employed (Fig. 7). The result indicated that sutureless technique, in comparison with with conventional surgery, was associated with a lower occurrence rate of PVO (OR, 0.52 95%CI, 0.32–0.86; *P* = 0.01).

**Table 4:** Post-operative PVO and strategy of re-operation

Study	Surgical approach	Post-operative PVO(n)	Strategy of post-operative PVO underwent re-operation	Total number of operation(n)
Gao [8]	ST CS	2 14	NA^a^	NA
Cui [9]	ST CS	NA	NA	NA
Shi [10]	ST CS	10 101	12 sutureless technique; 7 patch augmentation; 5 fibrous resection^b^	792^b^
Yamashita [11]	ST CS	1 2	1 sutureless technique 1 fibrous resection; 1 SI	8 8
Yanagawa [12]	ST CS	1 2	No patient required re-operation NA^c^	21 38
Honjo [13]	ST CS	1 6	No patient required re-operation 3 fibrous resection	8 17
Lo Rito [14]	ST CS	5 17	1 fibrous resection; 1 sutureless technique 5 fibrous resection; 7 sutureless technique	71 138

^a^ Only the numbers of PVO was available and lack of details of post-operative PVO

^b^ Strategy of post-operative PVO underwent re-operation was presented totally

^c^ Two patients in the conventional repair group underwent re-operation without description of details

*ST S*utureles technique group, *CS* Conventional surgery group, *SI* Stent implantation, *NA N*ot available

**Fig. 8:**
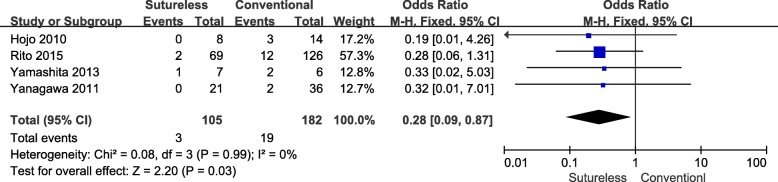
Forest plot of meta-analysis of occurrence rate of re-operation due to PVO

A total of 5 studies [10–14] analyzed the re-operation due to post-operative PVO of both surgeries (Table 4). However, since the Shi et al. [10] study only recorded the overall re-operation of both approaches but without particular data in each group, therefore, only 4 studies were included in this meta-analysis. A heterogeneity test revealed Chi^2^ = 0.08, *P* = 0.99, I^2^ = 0%, and a fixed effects model was employed (Fig. 8). This meta-analysis result indicated that sutureless technique, compared with conventional surgery, was associated with a lower occurrence rate of re-operation (OR, 0.28;95%CI, 0.09–0.87; *P* = 0.03).

All the re-operated patients covered by the 5 studies [10–14] had only one re-operation. Referred to Table 4 for the total number of operation.

### Sensitivity analysis

Sensitivity analyses using the leave-one-out method were adopted in the meta-analysis of CPB, aortic cross-clamp time and hospitalization time due to significant heterogeneity. In the meta-analysis of CPB and aortic cross-clamp time, the I^2^ was reduced to 0 and 11% respectively when the Gao et al. [8] study was removed, whereas the pooling estimates became statistically significant respectively at (WMD, 10.01;95%CI, 7.71–12.30; *P* < 0.001) and (WMD, 10.00;95%CI,7.77–12.24; *P* < 0.001). In the meta-analysis of hospitalization time, the I^2^ was reduced to 14% when the Yanagawa et al. [12] study was removed while the pooling estimate still remained statistically insignificant(WMD, 1.16; 95%CI, − 2.71-5.52; *P* = 0.50).

## Discussion

Sutureless technique, initially employed for the recurrent PVO, was subsequently used for primarily ‘prophylactic’ repair in TAPVC patients who were at high risk of developing post-operative PVO [4, 13, 15] after technique’s efficacy was proven and widely applied. The risk of developing post-operative PVO was particularly high for the following scenarios: the existence of preoperative hypoplastic pulmonary veins, young age at initial surgery, TAPVC patients with right atrial isomerism, cardiac TAPVC with preexisting PVO and mixed type TAPVC [1, 2, 13]. Preexisting PVO could be difficult to diagnose with cardiac echo. However, in our study, 6 out of 7 included studies (Table 1) reported the the detection of pre-operative PVO. So it was noteworthy that surgeons should consider sutureless technique as a prior alternative for the primary repair of TAPVC in such condition. Yaganawa et al. [12] and Honjo [13] reported the circumstances under which sutureless technique was used for the primary repair of TAPVC: 1) all infracardiac TAPVC; 2) cardiac TAPVC with stenosis in the pulmonary veins or in the short confluence bridging between the pulmonary veins and the coronary sinus or the right atrium; 3) surgeons’ preference for supracardiac TAPVC. Our study result was also consistent with that of previous studies in that it indicated sutureless technique entailed a lower occurrence rate of post-operative PVO (OR, 0.52) and re-operation (OR, 0.28). We found post-operative PVO was one of the main causes of death (Table 3). Therefore, sutureless technique should be recommended to patients with high risk of developing post-operative PVO. Even though sutureless technique was initially used for the recurrent PVO, surprisingly, the patch augmentation and fibrous resection were adopted as often as sutureless technique in the re-operation for PVO (Table 4) among our included studies. This practice could be due to the neoteric of the sutureless technique. A Cox proportional hazards analysis conducted by Yong et al. [16] suggested that operative weight < 2.5 kg and postoperative pulmonary hypertensive crisis were risk factors likely to trigger re-operation for post-operative PVO. However, the role of low body weight as a risk factor was far from conclusive as low body weight was found to be a high risk factor for every type of cardiac surgery. As such, the potentially complex interrelationship between low body weight and post-operative PVO warranted further investigation. But, tentatively, we postulated that relatively smaller pulmonary veins were generally associated with lower body weight. Therefore, to avoid distorsion of anastomosis in patients of low body weight with relatively smaller pulmonary veins, we suggested the use of sutureless technique in low weight infants with TAPVC.

The advantages for primary use of sutureless technique for TAPVC were obvious: 1) wide application to almost any type of pulmonary vein confluences, especially for patients with pulmonary vein stenosis [5, 13]; 2) better visualization of the edges of divided pulmonary veins to avoid Deep Hypothermic Circulatory Arrest (DHCA) [13, 15]; 3) avoided involving the vascular endothelium to reduce the scarring and distortion of pulmonary veins. The main disadvantage of the primary use of sutureless technique was potential bleeding from the gap between the pericardium and the confluence into the posterior mediastinum or pleural cavity. Yoshimura et al. [5] reported one case with intraoperative massive bleeding into the left pleural cavity. The four cases reported by Yun et al. [15] with similar complication could be managed by intrapleural hilar approximation. In our experience, as long as the integrity of the parietal pluera was kept intact, massive bleeding would not occur. The injury to the phrenic nerve in connection with sutureless technique was reported as well [13]. Other disadvantages such as thrombogenicity at the exposed pericardial surface, air embolism and soft tissue rupture were also reported [12]. Yaganawa et al. [12] reported primary sutureless repair to a neonate with mixed TAPVC involved the use of a distal incision into the individual pulmonary vein. Oscillation ventilation and ECMO were required postoperatively. And air bubbles found in the aorta during the ECMO initiation were probably related to the oscillation ventilation. However, we believe the air embolism was not caused by sutureless technique, but resulted from insufficient exhaust before weaning off CPB. In our center, we did not observe any patients with thrombogenicity or air embolism with the primary use of sutureless technique for TAPVC.

Sutureless technique enabled better visualization of pulmonary veins to avoid DHCA and also reduced the surgical exploration time of pulmonary veins. In addition, Yoshimura et al. [17] reported the suretureless technique was less technically demanding compared with conventional surgery. Therefore, the suretureless technique should theoretically reduce the CPB and aortic cross-clamp time. However, our study indicated that sutureless technique, contrary to conventional surgery, did not significantly reduce CPB and aortic cross-clamp time. In the meantime, in the sensitivity analysis of CPB and aortic cross-clamp time, we observed a significant heterogeneity reduction and the pooling estimates became statistically significant with the removal of the Gao et al. [8] study. It indicated that the meta-analysis of CPB and aortic cross-clamp time were overly influenced by the Gao et al. [8] study and the pooling results could be inconclusive. Compared with other included studies, the Gao et al. [8] study entailed a significantly longer CPB and aortic cross-clamp time in the conventional surgery group than that in the sutureless technique group. Longer CPB and aortic cross-clamp time might be explained by the complexity of the surgical procedure or unstable patients requiring longer CPB support. As expected, we observed a higher percentage of mixed TAPVC patients in their conventional surgery group compared with other studies and could contribute to their longer CPB and aortic cross-clamp time.

Hospitalization time was related to post-operative recovery. Meta-analysis of hospitalization time indicated that compared with conventional surgery, sutureless technique did not reduce the hospitalization time significantly. In the sensitivity analysis of hospitalization time, we observed a statistically significant reduction in heterogeneity while the pooling estimate still remained statistically insignificant upon the removal of the Yanagawa et al. [12] study. However, hospitalization time was largely due to the era effect based on the evolution in ICU management and preoperative diagnosis. Therefore more standard evaluation of post-operative recovery should be conducted. Other endpoints connected to post-operative recovery such as ICU stay and ventilation time were not available for pooling estimates due to lack of relevant data.

### Limitations

This was the first-ever study to-date to compare the outcomes between applying sutureless technique and conventional surgery for TAPVC patients. And several limitations to our study were identified as follows:

- First, all 7 studies included were retrospective NRCTs. Consequently, the level of evidence was low;
- Second, sutureless technique was generally adopted in higher risk patients of developing post-operative PVO. However, we only summarized the associated cardiac anomalies as baseline characteristics (Table 2) and failed to disclose the severity of preoperative PVO due to the divergent measurement method adopted in each study. Therefore, a high risk of selection bias of included patients might exist;
- Third, the endpoints that we evaluated such as mortality and hospitalization time were largely influenced by the era effect. The transition from conventional to primary sutureless repair represented the evolution of surgical techniques over time, which occurred inevitably in parallel with other evolution including but not limited to ICU management and preoperative diagnosis;
- Fourth, the only follow-up data we collected were post-operative PVO and mortality. The assessment of other long term outcomes of sutureless technique was absent in our study. The evaluation of cardiac-pulmonary function in the long term should have been included to augment the analysis of the benefits of each approach;
- Finally, we only conducted meta-analysis of sutureless technique in the primary repair of TAPVC, and no conclusion could be drawn whether sutureless technique was effective and safe in the repair of secondary PVO. Further meta-analysis should be conducted to explore the effectiveness and safey of sutureless technique for secondary PVO. Another limitation was that 2 out of 7 included studies were written in Chinese only, reducing their accessibility to thethe rest of the world.

## Conclusion

In conclusion, compared with conventional surgery, sutureless technique significantly reduced the occurrence rate of post-operative PVO and re-operation due to PVO. No significant statistical differences were observed between conventional surgery and sutureless technique with regard to mortality and hospitalization time. The initial meta-analysis of CPB and aortic cross-clamp time also found no statistical differences between the two surgical approaches. But the meta-analysis results could not be validated when highly heterogeneous studies were excluded following sensitivity analysis yielded different results. In terms of the occurrence rate of post-operative PVO and re-operation, our study showed sutureless technique was superior to conventional surgery for primary repair of TAPVC, especially for TAPVC patients who were at a high risk of developing post-operative PVO. The level of evidence of our study was low and RCTs should be designed to evaluate and compare the safety and effectiveness of primary sutureless technique for TAPVC.

## Abbreviations

CPB: Cardiopulmonary bypass time
ECMO: Extracorporeal membrane oxygenation
NOS: Newcastle-Ottawa Scale
NRCTs: None-randomized controlled trials
OR: Odds ratio
PVO: Pulmonary venous obstruction
TAPVC: Total anomalous pulmonary venous connection
WMD: Weighted mean difference
